# Screen Use and Mental Health Symptoms in Canadian Children and Youth During the COVID-19 Pandemic

**DOI:** 10.1001/jamanetworkopen.2021.40875

**Published:** 2021-12-28

**Authors:** Xuedi Li, Leigh M. Vanderloo, Charles D. G. Keown-Stoneman, Katherine Tombeau Cost, Alice Charach, Jonathon L. Maguire, Suneeta Monga, Jennifer Crosbie, Christie Burton, Evdokia Anagnostou, Stelios Georgiades, Rob Nicolson, Elizabeth Kelley, Muhammad Ayub, Daphne J. Korczak, Catherine S. Birken

**Affiliations:** 1 Child Health Evaluative Sciences, Peter Gilgan Centre for Research and Learning, The Hospital for Sick Children, Toronto, Ontario, Canada; 2 School of Occupational Therapy, Western University, London, Ontario, Canada; 3 ParticipACTION, Toronto, Ontario, Canada; 4 Li Ka Shing Knowledge Institute, St Michael’s Hospital, Toronto, Ontario, Canada; 5 Dalla Lana School of Public Health, University of Toronto, Toronto, Ontario, Canada; 6 Department of Psychiatry, The Hospital for Sick Children, Toronto, Ontario, Canada; 7 Department of Psychiatry, Faculty of Medicine, University of Toronto, Toronto, Ontario, Canada; 8 Department of Pediatrics, Faculty of Medicine, University of Toronto, Toronto, Ontario, Canada; 9 Department of Pediatrics, St Michael’s Hospital, Toronto, Ontario, Canada; 10 Holland Bloorview Research Institute, Toronto, Ontario, Canada; 11 Department of Psychiatry and Behavioural Neurosciences, McMaster University, Hamilton, Ontario, Canada; 12 Department of Psychiatry, Western University, London, Ontario, Canada; 13 Department of Psychology, Queens University, Kingston, Ontario, Canada; 14 Department of Psychiatry, Queens University, Kingston, Ontario, Canada

## Abstract

**Importance:**

Longitudinal research on specific forms of electronic screen use and mental health symptoms in children and youth during COVID-19 is minimal. Understanding the association may help develop policies and interventions targeting specific screen activities to promote healthful screen use and mental health in children and youth.

**Objective:**

To determine whether specific forms of screen use (television [TV] or digital media, video games, electronic learning, and video-chatting time) were associated with symptoms of depression, anxiety, conduct problems, irritability, hyperactivity, and inattention in children and youth during COVID-19.

**Design, Setting, and Participants:**

A longitudinal cohort study with repeated measures of exposures and outcomes was conducted in children and youth aged 2 to 18 years in Ontario, Canada, between May 2020 and April 2021 across 4 cohorts of children or youth: 2 community cohorts and 2 clinically referred cohorts. Parents were asked to complete repeated questionnaires about their children’s health behaviors and mental health symptoms during COVID-19.

**Main Outcomes and Measures:**

The exposure variables were children’s daily TV or digital media time, video game time, electronic-learning time, and video-chatting time. The mental health outcomes were parent-reported symptoms of child depression, anxiety, conduct problems and irritability, and hyperactivity/inattention using validated standardized tools.

**Results:**

This study included 2026 children with 6648 observations. In younger children (mean [SD] age, 5.9 [2.5] years; 275 male participants [51.7%]), higher TV or digital media time was associated with higher levels of conduct problems (age 2-4 years: β, 0.22 [95% CI, 0.10-0.35]; *P* < .001; age ≥4 years: β, 0.07 [95% CI, 0.02-0.11]; *P* = .007) and hyperactivity/inattention (β, 0.07 [95% CI, 0.006-0.14]; *P* = .04). In older children and youth (mean [SD] age, 11.3 [3.3] years; 844 male participants [56.5%]), higher levels of TV or digital media time were associated with higher levels of depression, anxiety, and inattention; higher levels of video game time were associated with higher levels of depression, irritability, inattention, and hyperactivity. Higher levels of electronic learning time were associated with higher levels of depression and anxiety.

**Conclusions and Relevance:**

In this cohort study, higher levels of screen use were associated poor mental health of children and youth during the COVID-19 pandemic. These findings suggest that policy intervention as well as evidence-informed social supports are needed to promote healthful screen use and mental health in children and youth during the pandemic and beyond.

## Key Points

**Question:**

What are the associations between different types of electronic screen use and mental health symptoms in Canadian children and youth during the COVID-19 pandemic?

**Findings:**

In this longitudinal study of 4 cohorts, 2026 children with 6648 observations were included. Compared with children with lower levels of screen use, children with higher levels of screen use had significantly higher levels of mental health symptoms during the COVID-19 pandemic.

**Meaning:**

These findings suggest that policy intervention, as well as evidence-informed social supports, may be required to promote healthful screen use and mental health in children and youth during the pandemic and beyond.

## Teaser

This cohort study examines whether specific forms of electronic screen use were associated with symptoms of depression, anxiety, conduct problems, irritability, hyperactivity, and inattention in children and youth during COVID-19.

## Introduction

The COVID-19 pandemic has resulted in major changes in the daily routines of children, primarily because of the imposed public health measures related to distancing, isolation, and school closures (see eAppendix 1 in the Supplement for COVID-19 public health measures and daily cases in Ontario, Canada).^1,2^ Children and youth reported higher levels of electronic screen use during the pandemic compared with prepandemic levels.^3,4,5,6,7,8^ Our group reported that children’s adherence to COVID-19 public health measures was associated with higher screen use among young children in Ontario.^9^

Research^10,11,12^ conducted before the pandemic consistently showed that high levels of screen use are associated with depression, anxiety, conduct disorders, and attention problems in children and youth, although causality cannot be concluded. Research^13^ by our group provided evidence that stress due to social isolation was associated with deterioration in multiple mental health domains during COVID-19. In addition to high screen use and social isolation, the worsening of child mental health could be related to the displacement of sleep, physical exercise, and other prosocial activities, which were disrupted during the pandemic.^3,5,14^ The exposure to online bullying, stressful news, and harmful advertisements during screen use could also contribute to poor child mental health during the pandemic.^15^

There is consistent evidence that the types of screen use (eg, television [TV] viewing, video game playing, and computer use) modify the association with mental health symptoms.^16^ Evidence on the roles of child age and sex in this association is inconsistent.^16^ Besides TV or digital media use and video gaming, video chatting and electronic learning have become more prominent during the pandemic. Video chatting has been encouraged to stay socially connected while practicing physical distancing,^17^ and electronic learning has become an important mode of education in Ontario because of the prolonged school closures.^2^ Longitudinal research on the associations of these multiple types of screen use with mental health symptoms in children and youth during the pandemic is sparse.

Children with autism spectrum disorder (ASD) are engaged in more screen use than typically developing children and other clinical groups.^18^ They also show earlier preference and greater affinity for technology, engaging with screens at an earlier age.^19^ Therefore, children with ASD may have entered the pandemic with higher screen use than their peers. In addition, because children with ASD have impaired social skills and engage in fewer social interactions,^20^ with the reduction of social interactions and replacing of prosocial activities with screen use during the pandemic, children with ASD might not have experienced the same degree of worsening mental health compared with typically developing children during the pandemic. Research examining screen use and mental health in children with ASD during the pandemic is minimal.

Understanding the association of different types of screen use with child and youth mental health may help inform the development of policies and interventions to promote healthful screen use and mental health in children and youth during the pandemic and beyond. The primary objective was to determine whether specific forms of screen use (TV or digital media, video game, electronic learning, and video chatting) were associated with child and youth mental health symptoms (depression, anxiety, conduct problems and irritability, hyperactivity, and inattention) during COVID-19. The secondary objective was to explore whether these associations differed by child sex and age. We aimed to explore whether the associations were different for children with a diagnosis of ASD. We hypothesized that high levels of TV or digital media time and video game time would be associated with poorer mental health status, with higher risk among male and older children and a minimal association for children with ASD. We hypothesized that higher levels of electronic-learning and video-chatting time would be associated with improved mental health outcomes as a result of direct social interaction.

## Methods

### Study Design and Participants

A longitudinal study using repeated measures of exposures and outcomes was conducted in Ontario, Canada, between May 2020 and April 2021 in 4 ongoing study cohorts (2 community cohorts and 2 clinically referred mental health and neurodevelopmental disorder cohorts).^13^ The Applied Research Group for Kids (TARGetKids!) is a practice-based, primary care research network, enrolling healthy children aged 0 to 5 years from primary health care settings in the Greater Toronto Area.^21^ Spit for Science is a population-based sample of children aged 6 to 18 years recruited at the Ontario Science Centre, a science museum in Toronto.^22,23^ SickKids Psychiatry enrolled children aged 6 to 18 years in the Greater Toronto Area referred to an outpatient mental health clinic for evaluation of mental health concerns, including but not limited to depression and anxiety disorders, attention-deficit/hyperactivity disorder (ADHD), and obsessive-compulsive disorder (OCD) (see eAppendix 2 in the Supplement for cohort details). Finally, the Province of Ontario Neurodevelopmental Disorder Network (POND) enrolled children aged 6 to 18 years in Ontario receiving care at outpatient clinics with neurodevelopmental disorders, including ASD.^24,25^ All participating families provided informed written consent, and this study was approved by all affiliated research ethics boards. This study follows the Strengthening the Reporting of Observational Studies in Epidemiology (STROBE) reporting guideline for cohort studies.

The validated standardized tool to assess mental health symptoms in TARGetKids! was different from the ones used in the 3 other cohorts because of the younger age group of the participants. Therefore, we performed analyses separately for the TARGetKids! cohort and the other cohorts. We only included observations with exposure measures collected before or at the same time as the outcome measures.

Questionnaires were administered repeatedly to participating parents during COVID-19. Parents participating in the 4 cohorts were invited via email or telephone to complete questionnaires online about physical and mental health of children and parents, health behaviors including screen use, and sociodemographic information. Recruitment for each cohort started in April 2020 (first wave of COVID-19 in Ontario) and is ongoing throughout the pandemic. All invited participants were sent email reminders and phone call reminders as methods of follow-up. A total of 138 participants withdrew from this study between May 2020 and April 2021.

### Exposures

The exposures in this study were parent-reported child daily TV and digital media time, video game time, electronic-learning time, and video-chatting time (questions shown in eTable 1 in the Supplement) collected between May 21, 2020, and April 9, 2021. For younger children (TARGetKids! cohort), daily screen time duration was measured continuously and collected every other week. For older children and youth (the other 3 cohorts), the child’s daily screen use measures were collected monthly and responses were categorical with 6 response options: 0 to 30 minutes, 1 hour, 2 to 3 hours, 4 to 5 hours, 6 to 8 hours, and 9 hours or more.

### Outcomes

The outcome variables in all 4 cohorts were parent-reported child and youth mental health symptom scales measured in multiple domains during the pandemic. The primary outcome was depression and anxiety. The secondary outcomes were conduct problems, irritability, hyperactivity, and inattention. In younger children, the 5 subscales of Strengths and Difficulties Questionnaire (SDQ)^26,27,28^ was administered monthly to parents. The SDQ emotional symptoms subscale was used to assess depression and anxiety, the conduct problems subscale was used to assess conduct problems (using 2 age-dependent versions for children aged 24 months to <48 months [referred to in the SDQ as 2-4 years] and those aged ≥48 months [referred to in the SDQ as 4-17 years]),^29,30^ and the hyperactivity/inattention subscale was used to assess hyperactivity and inattention (2 age-dependent versions for children aged 2-4 years and those aged 4-17 years).

For older children and youth, the following validated parent-reported tools were used. To assess depression, the 10-item major depressive disorder subscale of RCADS-P (Revised Children’s Anxiety and Depression Scale–Parent Version) T-score was used.^31,32^ To assess anxiety, the 9-item Generalized Anxiety Disorder subscale from the SCARED (Screen for Child Anxiety Related Disorders) was used.^33^ RCADS-P and SCARED were measured monthly from May 2020 to April 2021. The 6-item subscale from TIDES (The Irritability and Dysregulation of Emotions Questionnaire) was administered to measure irritability 3 times in May and November 2020 and February 2021. To measure inattention and hyperactivity, the 18-item total score, the 9-item inattentive subscale, and the hyperactive/impulsive subscale of the SWAN (Strengths and Weaknesses of Attention-Deficit/Hyperactivity Disorder Symptoms and Normal Behavior Scale)^34,35^ were administered in November 2020 and February 2021. For all the aforementioned scales, higher scores are indicative of greater numbers of symptoms.

### Covariates

Potential confounders identified a priori included child age measured at the time of mental health outcomes, child sex, race and ancestry, self-reported annual family income, previous ASD diagnosis, and calendar date. Maternal race and ancestry were collected in TARGetKids! cohort, and child race and ancestry were collected in the other 3 cohorts. Race and ancestry were assessed in this study because they may be associated with both the exposure and the outcomes. Calendar date was the date when the screen use questionnaire was completed and was used to capture other time-varying external changes, such as changing public health guidelines. We used restricted cubic splines with 5 knots to accommodate various shapes for the association of calendar date with the outcome in the models. Parental mental health status during COVID-19 was included as a variable associated with the outcome in the model measured by Generalized Anxiety Disorder–7 (GAD-7)^36^ and the Patient Health Questionnaire (PHQ-8).^37^ Higher scores in GAD-7 or PHQ-8 were indicative of greater symptoms. GAD-7 was collected in May 2020 in all 4 cohorts. PHQ-8 was collected every other week in TARGetKids! and once in May 2020 in the other 3 cohorts. Parent-reported child previous diagnosis of mental health conditions (depression, anxiety, OCD, and ADHD) was considered a confounder in a post hoc analysis with the 3 cohorts. Child age, sex, and previous ASD diagnosis were determined as potential interactions with the exposures a priori.

### Statistical Analysis

Separate analyses were performed to accommodate the different measures used in the different age groups. Linear mixed-effects models were fitted using repeated measures of exposures and outcomes, including random intercepts for the families and participants within families, because this study included siblings from the same family (see eAppendix 3 in the Supplement for model description). An unadjusted model and an adjusted model were fitted to assess the association between each screen type and each mental health domain. We performed a post hoc exploratory analysis also adjusting for previous diagnosis of mental health conditions in the 3 cohorts of older children and youth. Likelihood ratio tests were used to assess the evidence that child age, sex, or previous ASD diagnosis modified the associations between the exposures and the outcomes. We assumed that data were missing at random conditional on the other variables included in the model and that the probability of withdrawal was unrelated to the association between screen use and mental health. Multivariate imputation with chained equations (using 15 imputed data sets) was performed using the mice package in R to account for potential bias introduced from missing covariates data.^38^ All *P* values were 2-tailed, and statistical significance was set at α = .05. R statistical software version 4.0.2 (R Project for Statistical Computing) was used for all analyses.^39^

## Results

This study included 2026 children with 6648 total observations (see eFigure 1 in the Supplement for sample size flowchart). A total of 532 children with 1962 observations were included in the TARGetKids! cohort. Participant characteristics are presented in Table 1: the mean (SD) age was 5.9 (2.5) years, 275 children (51.7%) were male, and 319 (68.9%) had mothers of European ancestry. For the other 3 cohorts combined, a total of 1494 children with 4686 observations were included. The mean (SD) age was 11.3 (3.3) years, 844 children (56.5%) were male, and 843 (57.5%) were of European ancestry. Two hundred thirty-seven children (15.9%) had a previous diagnosis of ASD, and 785 (52.5%) had a previous diagnosis of any mental health condition. Screen use and mental health outcomes are shown in Table 2. Data for screen use and mental health outcomes over time are shown in eFigure 2, eFigure 3, eFigure 4, and eFigure 5 in the Supplement.

**Table 1.** Participant Characteristics

Characteristic	Participants, No. (%)
Younger children (TARGetKids! cohort) (n = 532 [0% missing])	
Child age, mean (SD), y	5.9 (2.5)
<4	140 (26.3)
≥4	392 (73.7)
Child sex (n = 532 [0% missing])	
Male	275 (51.7)
Female	257 (48.3)
Maternal race or ancestry (n = 463 [13.0% missing])	
Black	10 (2.2)
East Asian	45 (9.7)
European	319 (68.9)
South and Southeast Asian	38 (8.2)
Multiple	32 (6.9)
Other^a^	19 (4.1)
Self-reported annual household income, $ (n = 504 [5.3% missing])	
0-39 999	13 (2.6)
40 000-79 999	59 (11.7)
80 000-149 999	154 (30.6)
≥150 000	278 (55.2)
Previous diagnosis of ASD (n = 492 [7.3%] missing)	
Yes	9 (1.8)
No	483 (98.2)
Parent GAD-7 score, mean (SD) (n = 467 [12.2%] missing)	5.6 (4.4)
Parent PHQ-8 score, mean (SD) (n = 467 [12.2%] missing)	4.1 (4.5)
Older children (Spit for Science, SickKids Psychiatry, and POND cohorts) (n = 1494)	
Child age, mean (SD), y (n = 1494 [0%] missing)	11.3 (3.3)
Child sex (n = 1494 [0%] missing)	
Male	844 (56.5)
Female	650 (43.5)
Self-reported annual household income, $ (n = 1404 [6.0%] missing)	
<29 999	91 (6.5)
30 000-49 999	92 (6.6)
50 000-74 999	183 (13.0)
80 000-99 999	167 (11.9)
100 000-199 999	473 (33.7)
>200 000	249 (17.7)
Choose not to answer	149 (10.6)
Child ancestry (n = 1467 [1.8%] missing)	
European	843 (57.5)
Non-European	268 (18.3)
Multiple	356 (24.3)
Previous diagnosis of ASD (n = 1494 [0%] missing)	
No	1257 (84.1)
Yes	237 (15.9)
Previous diagnosis of depression (n = 1494 [0%] missing)	
No	1358 (90.9)
Yes	136 (9.1)
Previous diagnosis of anxiety (n = 1494 [0%] missing)	
No	918 (61.4)
Yes	576 (38.6)
Previous diagnosis of OCD (n = 1494 [0%] missing)	
No	1383 (92.6)
Yes	111 (7.4)
Previous diagnosis of ADHD (n = 1494 [0%] missing)	
No	1072 (71.8)
Yes	422 (28.2)
Previous diagnosis of any mental health conditions (n = 1494 [0%] missing)^b^	
No	709 (47.5)
Yes	785 (52.5)
Parent, mean (SD)	
GAD-7 (n = 1392 [6.8%] missing)	6.7 (5.1)
PHQ-8 (n = 1392 [6.8%] missing)	7.5 (5.5)

Abbreviations: ASD, Autism Spectrum Disorder; ADHD, attention-deficit/hyperactivity disorder; OCD, obsessive-compulsive disorder; GAD-7, Generalized Anxiety Disorder Scale; PHQ-8, Patient Health Questionnaire.

^a^ Other includes Indigenous, Latinx, and Middle Eastern ancestries.

^b^ Mental health conditions include depression, anxiety, OCD, and ADHD.

**Table 2.** Parent-Reported Child Daily Screen Use and Mental Health Outcomes During the COVID-19 Pandemic From May 21, 2020, to April 9, 2021

Child daily screen use and mental health outcomes	Mean (SD)
Younger children (TARGetKids! cohort)	
Child daily screen use time	
Watching television or digital media (n = 1962 observations)	
Minutes per day	97.0 (78.9)
Hours per day	1.6 (1.3)
Playing video games (n = 1873 observations)	
Minutes per day	25.9 (47.4)
Hours per day	0.4 (0.8)
Electronic learning or online schoolwork (n = 1859 observations)	
Minutes per day	37.0 (84.9)
Hours per day	0.6 (1.4)
Video chatting or face-to-face communication (n = 1871 observations)	
Minutes per day	16.5 (44.3)
Hours per day	0.3 (0.7)
Child mental health outcomes scores	
Strength and Difficulties Questionnaire problems subscale	
Emotional (n = 1962 observations)	1.7 (1.8)
Conduct (n = 1953 observations)	
Version, y	
2-4 (n = 460 observations)	2.1 (1.7)
4-17 (n = 1493 observations)	1.4 (1.4)
Strength and Difficulties Questionnaire hyperactivity/inattention subscale (n = 1957 observations)	
Version, y	
2-4 (n = 461 observations)	3.6 (2.1)
4-17 (n = 1496 observations)	3.2 (2.7)
Older children (Spit for Science, SickKids Psychiatry, and POND cohorts)^a^	
Child daily screen use time, participants, No. (%)	
0-30 min	
Watching TV or digital media	275 (5.9)
Playing video games	1598 (34.1)
Electronic learning or online schoolwork	1768 (37.7)
Video chatting or face-to-face communication	2084 (63.8)
1 h	
Watching TV or digital media	796 (17.0)
Playing video games	1023 (21.8)
Electronic learning or online schoolwork	777 (16.6)
Video chatting or face-to-face communication	659 (20.2)
2-3 h	
Watching TV or digital media	1818 (38.8)
Playing video games	1232 (26.3)
Electronic learning or online schoolwork	907 (19.4)
Video chatting or face-to-face communication	351 (10.8)
4-5 h	
Watching TV or digital media	1026 (21.9)
Playing video games	505 (10.8)
Electronic learning or online schoolwork	696 (14.9)
Video chatting or face-to-face communication	102 (3.1)
6-8 h	
Watching TV or digital media	513 (10.9)
Playing video games	214 (4.6)
Electronic learning or online schoolwork	465 (9.9)
Video chatting or face-to-face communication	49 (1.5)
≥9 h	
Watching TV or digital media	258 (5.5)
Playing video games	114 (2.4)
Electronic learning or online schoolwork	73 (1.6)
Video chatting or face-to-face communication	20 (0.6)
Child mental health outcomes	
RCADS-P T-score (n = 4686 observations)	60.2 (17.4)
SCARED (n = 4686 observations)	7.1 (5.3)
TIDES (n = 3243 observations)	0.4 (8.5)
SWAN (n = 1831 observations)	
Total score	3.2 (21.9)
Inattentive subscale	2.9 (11.8)
Hyperactive/impulsive subscale	0.3 (11.3)

Abbreviations: RCADS-P, Revised Children’s Anxiety and Depression Scale–Parent Version; SCARED, Screen for Child Anxiety Related Disorders; SWAN, Strengths and Weaknesses of Attention-Deficit/Hyperactivity Disorder Symptoms and Normal Behavior Scale; TIDES, The Irritability and Dysregulation of Emotions Questionnaire; TV, television.

^a^ There were 4686 observations each for watching TV or digital media, playing video games, and electronic learning or online schoolwork and 3265 observations for video chatting or face-to-face communication.

### Association of Screen Use With Mental Health Domains in Younger Children (TARGetKids! Cohort)

In the analyses for TARGetKids! (Table 3), it was estimated that every additional hour per day that children watched TV or digital media was associated with a higher mean SDQ conduct problems score of 0.22 in the adjusted model in children aged 2 to 4 years (95% CI, 0.10-0.35; *P* < .001) and of 0.07 in the adjusted model in children aged 4 years and older (95% CI, 0.02-0.11; *P* = .007). Every additional hour per day that children watched TV or digital media was associated with a higher mean SDQ hyperactivity/inattention score of 0.07 in the adjusted model in children aged 4 years and older (95% CI, 0.006-0.14; *P* = .04). There was insufficient evidence of associations between the other types of screen use and SDQ subscales.

**Table 3.** Association of Screen Use With Child Mental Health Domains in Younger Children (TARGetKids! Cohort) During COVID-19 From May 21, 2020, to April 9, 2021

Domain and type of screen use	Unadjusted		Adjusted^a^	
	β (95% CI)	*P* value	β (95% CI)	*P* value
Depression-anxiety (SDQ emotional symptoms subscale)^b^				
TV or digital media	0.03 (−0.02 to 0.08)	.29	−0.006 (−0.06 to 0.05)	.82
Video game	−0.007 (−0.09 to 0.09)	.99	−0.02 (−0.13 to 0.10)	.76
Electronic learning	0.04 (0.002 to 0.08)	.04	0.02 (−0.02 to 0.07)	.29
Video chatting	−0.01 (−0.09 to 0.06)	.70	−0.03 (−0.10 to 0.05)	.48
Conduct problems (SDQ conduct problems subscale, version 2-4 y)				
TV or digital media	0.25 (0.14 to 0.36)	<.001	0.22 (0.10 to 0.35)	<.001
Video game	0.01 (−0.38 to 0.40)	.96	−0.11 (−0.51 to 0.30)	.60
Electronic learning	−0.007 (0.24 to 0.22)	.96	−0.04 (−0.28 to 0.20)	.73
Video chatting	0.03 (−0.07 to 0.13)	.57	0.03 (−0.07 to 0.14)	.54
Conduct problems (SDQ conduct problems subscale, version 4-17 y)				
TV or digital media	0.09 (0.05 to 0.14)	<.001	0.07 (0.02 to 0.11)	.007
Video game	0.07 (−0.008 to 0.15)	.08	0.06 (−0.02 to 0.14)	.17
Electronic learning	0.01 (−0.02 to 0.05)	.31	0.02 (−0.02 to 0.05)	.28
Video-chatting	0.07 (−0.02 to 0.17)	.15	0.06 (−0.04 to 0.15)	.24
Hyperactivity/inattention (SDQ hyperactivity/inattention subscale, version 2-4 y)				
TV or digital media	−0.03 (−0.16 to 0.10)	.63	−0.06 (−0.21 to 0.08)	.37
Video game	0.02 (−0.39 to 0.44)	.92	0.04 (−0.39 to 0.46)	.87
Electronic learning	0.004 (−0.24 to 0.25)	.98	−0.02 (−0.27 to 0.24)	.90
Video chatting	−0.006 (−0.11 to 0.10)	.92	−0.009 (−0.12 to 0.10)	.88
Hyperactivity/inattention (SDQ hyperactivity/inattention subscale, version 4-17 y)				
TV or digital media	0.08 (0.02 to 0.15)	.01	0.07 (0.006 to 0.14)	.04
Video game	0.08 (−0.04 to 0.20)	.17	0.04 (−0.08 to 0.17)	.4
Electronic learning	0.07 (0.02 to 0.12)	.004	0.04 (−0.007 to 0.10)	.09
Video chatting	0.12 (−0.02 to 0.25)	.09	0.12 (−0.02 to 0.26)	.10

Abbreviations: SDQ, Strength and Difficulties Questionnaire; TV, television.

^a^ Adjusted for child age, child sex, maternal ancestry, self-reported family income, previous autism spectrum disorder diagnosis, calendar date, Generalized Anxiety Disorder Scale–7 score, and Patient Health Questionnaire–8 score.

^b^ The Bonferroni-corrected α is .0125 after adjusting for multiple (4) comparisons.

### Association of Screen Use With Mental Health Domains in Older Children (Spit for Science, SickKids Psychiatry, and POND Cohorts)

Because screen use was a categorical variable with 6 levels in older children, we report here the effect sizes and 95% CIs for TV or digital media time and depression as an example because of space limitations. For the rest of the models, we report the trends from the adjusted models (effect sizes and 95% CIs for each level are reported in Table 4 and Table 5). Higher TV or digital media time per day was associated with higher levels of depression symptoms (1 hour: β, 0.21 [95% CI, −1.28 to 0.78]; 2-3 hours: β, 1.81 [95% CI, 0.29 to 3.33]; 4-5 hours: β, 2.80 [95% CI, 1.15 to 4.44]; 6-8 hours: β, 5.16 [95% CI, 3.32 to 7.01]; ≥9 hours: β, 5.42 [95% CI, 3.30 to 7.54]; overall *P* < .001). Similarly, higher TV or digital media time per day was associated with higher levels of anxiety symptoms. As there was evidence to suggest that previous ASD diagnosis modified the association with depression, the analyses were therefore stratified by ASD diagnosis (eTable 2 in the Supplement). The trend of a positive association with depression persisted in children without an ASD diagnosis but was not evident in those with ASD. TV or digital media time per day was also significantly associated with differences in symptoms of irritability, inattention, and hyperactivity/inattention. There was sufficient evidence that TV or digital media time was associated with hyperactivity/impulsivity; the association appears to be potentially nonlinear, with the lowest estimated mean SWAN hyperactivity/impulsive subscale at 2 to 3 hours of TV or digital media time.

**Table 4.** Association of TV or Digital Media Time and Video Game Time With Child Mental Health Domains in Older Children (Spit for Science, SickKids Psychiatry and POND Cohorts) During COVID-19 From May 21, 2020, to April 9, 2021^a^

Child screen use, h/d	TV or digital media						Video game					
	Unadjusted		Adjusted^b^		Post hoc adjusted^c^		Unadjusted		Adjusted^b^		Post hoc adjusted^c^	
	β (95% CI)	*P* value	β (95% CI)	*P* value	β (95% CI)	*P* value	β (95% CI)	*P* value	β (95% CI)	*P* value	β (95% CI)	*P* value
Depression (RCADS-P T-score)^d^												
1 h	0.56 (−0.97 to 2.09)	<.001	0.21 (−1.28 to 0.78)	<.001	0.14 (−1.34 to 1.63)	<.001	0.90 (−0.08 to 1.88)	<.001	0.81 (−0.15 to 1.77)	<.001	0.74 (−0.21 to 1.69)	<.001
2-3 h	2.66 (1.12 to 4.21)		1.81 (0.29 to 3.33)		1.69 (0.18 to 3.19)		3.45 (2.39 to 4.51)		2.73 (1.69 to 3.78)		2.54 (1.51 to 3.57)	
4-5 h	4.28 (2.63 to 5.92)		2.80 (1.15 to 4.44)		2.59 (0.96 to 4.21)		5.71 (4.33 to 7.09)		4.64 (3.27 to 6.02)		4.30 (2.94 to 5.66)	
6-8 h	6.87 (5.05 to 8.69)		5.16 (3.32 to 7.01)		4.90 (3.07 to 6.72)		8.23 (6.37 to 10.09)		6.73 (4.89 to 8.57)		6.28 (4.46 to 8.09)	
≥9 h	7.46 (5.36 to 9.57)		5.42 (3.30 to 7.54)		5.08 (2.00 to 7.18)		8.18 (5.66 to 10.70)		5.99 (3.48 to 8.50)		5.17 (2.69 to 7.64)	
Anxiety (SCARED score)^d^												
1 h	0.09 (−0.33 to 0.51)	<.001	0.10 (−0.32 to 0.52)	.003	0.07 (−0.35 to 0.49)	.01	0.03 (−0.24 to 0.31)	.89	0.10 (−0.18 to 0.37)	.90	0.08 (−0.19 to 0.35)	.92
2-3 h	0.34 (−0.09 to 0.76)		0.33 (−0.10 to 0.75)		0.29 (−0.14 to 0.71)		0.16 (−0.14 to 0.46)		0.18 (−0.12 to 0.48)		0.12 (−0.17 to 0.42)	
4-5 h	0.54 (0.09 to 0.99)		0.48 (0.01 to 0.95)		0.41 (−0.06 to 0.87)		0.15 (−0.23 to 0.54)		0.17 (−0.23 to 0.56)		0.04 (−0.35 to 0.43)	
6-8 h	0.95 (0.45 to 1.45)		0.90 (0.37 to 1.42)		0.80 (0.29 to 1.32)		0.07 (−0.45 to 0.59)		0.04 (−0.48 to 0.57)		−0.10 (−0.61 to 0.42)	
≥9 h	0.74 (0.16 to 1.32)		0.66 (0.06 to 1.26)		0.53 (−0.06 to 1.13)		0.26 (−0.44 to 0.97)		0.20 (−0.52 to 0.92)		−0.03 (−0.74 to 0.67)	
Irritability (TIDES score)												
1 h	−0.32 (−1.79 to 1.16)	<.001	−0.42 (−1.88 to 1.04)	.001	−0.23 (−1.60 to 1.15)	.007	0.92 (0.16 to 1.67)	<.001	0.97 (0.21 to 1.73)	.04	0.69 (−0.03 to 1.40)	.18
2-3 h	0.45 (−0.95 to 1.84)		0.23 (−1.15 to 1.61)		0.25 (−1.06 to 1.56)		0.88 (0.11 to 1.65)		0.87 (0.09 to 1.65)		0.40 (−0.33 to 1.13)	
4-5 h	1.22 (−0.22 to 2.67)		0.79 (−0.65 to 2.23)		0.71 (−0.65 to 2.07)		1.90 (0.90 to 2.91)		1.72 (0.69 to 2.76)		1.19 (0.22 to 2.15)	
6-8 h	1.75 (0.21 to 3.29)		1.18 (−0.36 to 2.72)		1.08 (−0.38 to 2.54)		2.27 (0.91 to 3.64)		1.93 (0.54 to 3.32)		0.72 (−0.57 to 2.00)	
≥9 h	2.67 (1.01 to 4.33)		2.22 (0.56 to 3.89)		1.92 (0.35 to 3.50)		2.39 (0.64 to 4.15)		2.24 (0.46 to 4.03)		1.13 (−0.53 to 2.78)	
Inattention/hyperactivity (SWAN total score)												
1 h	2.04 (−1.82 to 5.90)	.007	1.31 (−2.42 to 5.05)	.009	0.88 (−2.78 to 4.55)	.02	2.29 (0.22 to 4.36)	<.001	1.62 (−0.39 to 3.62)	.03	1.26 (−0.70 to 3.23)	.13
2-3 h	1.11 (−2.54 to 4.77)		0.76 (−2.79 to 4.30)		0.37 (−3.10 to 3.85)		3.97 (1.83 to 6.10)		2.15 (0.07 to 4.24)		1.54 (−0.49 to 3.58)	
4-5 h	3.88 (0.08 to 7.68)		3.78 (0.07 to 7.49)		3.08 (−0.56 to 6.72)		5.73 (2.88 to 8.57)		3.89 (1.09 to 6.70)		3.15 (0.42 to 5.88)	
6-8 h	2.77 (−1.32 to 6.87)		3.22 (−0.81 to 7.25)		2.58 (−1.37 to 6.54)		8.61 (4.72 to 12.50)		5.45 (1.61 to 9.29)		4.27 (0.50 to 8.04)	
≥9 h	5.63 (1.21 to 10.06)		4.61 (0.22 to 9.00)		4.13 (−0.17 to 8.43)		9.90 (4.90 to 14.89)		6.25 (1.34 to 11.16)		4.95 (0.17 to 9.73)	
Inattention (SWAN inattentive subscale)												
1 h	1.95 (−0.23 to 4.12)	.003	1.54 (−0.58 to 3.65)	.02	1.27 (−0.30 to 3.34)	.04	1.04 (−0.12 to 2.20)	<.001	0.74 (−0.40 to 1.87)	.03	0.54 (−0.57 to 1.65)	.13
2-3 h	1.59 (−0.47 to 3.65)		1.26 (−0.57 to 3.26)		1.01 (−0.95 to 2.97)		2.26 (1.07 to 3.45)		1.32 (0.14 to 2.49)		0.98 (−0.17 to 2.12)	
4-5 h	2.88 (0.74 to 5.02)		2.58 (0.47 to 4.68)		2.15 (0.09 to 4.21)		3.34 (1.76 to 4.93)		2.26 (0.68 to 3.84)		1.83 (0.29 to 3.36)	
6-8 h	2.56 (0.25 to 4.86)		2.43 (0.15 to 4.71)		2.01 (−0.22 to 4.25)		4.70 (2.52 to 6.88)		2.79 (0.62 to 4.96)		2.09 (−0.03 to 4.22)	
≥9 h	4.38 (1.89 to 6.87)		3.43 (0.95 to 5.91)		3.11 (0.68 to 5.54)		6.11 (3.32 to 8.90)		3.71 (0.94 to 6.48)		2.95 (0.25 to 5.64)	
Hyperactivity/impulsivity (SWAN hyperactive/impulsive subscale)												
1 h	0.08 (−2.02 to 2.17)	.04	−0.30 (−2.33 to 1.73)	.01	−0.49 (−2.48 to 1.51)	.03	1.33 (0.21 to 2.45)	<.001	0.92 (−0.17 to 2.00)	.04	0.74 (−0.33 to 1.80)	.21
2-3 h	−0.36 (−2.34 to 1.63)		−0.47 (−2.39 to 1.45)		−0.64 (−2.53 to 1.25)		1.97 (0.82 to 3.11)		0.94 (−0.18 to 2.06)		0.62 (−0.48 to 1.72)	
4-5 h	1.17 (−0.90 to 3.23)		1.23 (−0.78 to 3.25)		0.90 (−1.07 to 2.88)		2.74 (1.21 to 4.26)		1.77 (0.27 to 3.28)		1.40 (−0.07 to 2.87)	
6-8 h	0.57 (−1.65 to 2.79)		0.97 (−1.21 to 3.16)		0.67 (−1.48 to 2.81)		4.50 (2.41 to 6.60)		2.89 (0.82 to 4.96)		2.30 (0.26 to 4.34)	
≥9 h	1.53 (−0.87 to 3.94)		1.22 (−1.15 to 3.60)		1.01 (−1.33 to 3.35)		4.48 (1.79 to 7.16)		2.86 (0.23 to 5.50)		2.18 (−0.40 to 4.76)	

Abbreviations: ASD, autism spectrum disorder; GAD-7, Generalized Anxiety Disorder Scale; PHQ-8, Patient Health Questionnaire; RCADS-P, Revised Children’s Anxiety and Depression Scale–Parent Version; SCARED, Screen for Child Anxiety Related Disorders; SWAN, Strengths and Weaknesses of Attention-Deficit/Hyperactivity Disorder Symptoms and Normal Behavior Scale; TIDES, The Irritability and Dysregulation of Emotions Questionnaire; TV, television.

^a^ The reference group was 0 to 30 minutes per day.

^b^ Adjusted for child age (measured at mental health outcomes), child sex, child ancestry, family income, previous ASD diagnosis, calendar date, GAD-7, and PHQ-8.

^c^ Adjusted for child age (measured at mental health outcomes), child sex, child ancestry, family income, previous ASD diagnosis, calendar date, GAD-7, PHQ-8, and previous mental health (depression, anxiety, obsessive compulsive disorder, and attention-deficit/hyperactivity disorder) diagnosis.

^d^ The Bonferroni-corrected α is .0125 after adjusting for multiple (4) comparisons.

**Table 5.** Association of Electronic Learning Time and Video Chatting Time With Child Mental Health Domains in Older Children (Spit for Science, SickKids Psychiatry, and POND Cohorts) During COVID-19 From May 21, 2020, to April 9, 2021^a^

Child screen use, h/d	Electronic learning						Video chatting					
	Unadjusted		Adjusted^b^		Post hoc adjusted^c^		Unadjusted		Adjusted^b^		Post hoc adjusted^c^	
	β (95% CI)	*P* value	β (95% CI)	*P* value	β (95% CI)	*P* value	β (95% CI)	*P* value	β (95% CI)	*P* value	β (95% CI)	*P* value
Depression (RCADS-P T-score)^d^												
1 h	0.82 (−0.08 to 1.72)	<.001	0.24 (−0.71 to 1.18)	.004	0.27 (−0.67 to 1.21)	.005	0.88 (−0.14 to 1.89)	<.001	0.17 (−0.84 to 1.19)	.03	0.23 (−0.77 to 1.24)	.03
2-3 h	2.08 (1.23 to 2.93)		0.80 (−0.14 to 1.74)		0.83 (−0.10 to 1.76)		2.26 (0.86 to 3.66)		0.94 (−0.44 to 2.32)		0.90 (−0.46 to 2.27)	
4-5 h	3.04 (2.10 to 3.98)		1.41 (0.36 to 2.45)		1.46 (0.42 to 2.50)		2.67 (0.30 to 5.04)		0.96 (−1.35 to 3.26)		0.82 (−1.48 to 3.13)	
6-8 h	4.19 (3.06 to 5.31)		2.47 (1.20 to 3.74)		2.39 (1.14 to 3.64)		6.80 (3.43 to 10.17)		5.48 (2.21 to 8.75)		5.50 (2.28 to 8.72)	
≥9 h	2.37 (−0.18 to 4.91)		1.21 (−1.34 to 3.76)		1.06 (−1.47 to 3.59)		5.00 (−0.32 to 10.32)		2.15 (−3.02 to 7.32)		1.70 (−3.39 to 6.79)	
Anxiety (SCARED score)^d^												
1 h	−0.07 (−0.32 to 0.18)	<.001	0.06 (−0.20 to 0.33)	.002	0.08 (−0.19 to 0.34)	.001	0.25 (−0.02 to 0.53)	.008	0.20 (−0.09 to 0.49)	.11	0.21 (−0.07 to 0.50)	.12
2-3 h	0.32 (0.08 to 0.55)		0.40 (0.14 to 0.67)		0.42 (0.16 to 0.68)		0.70 (0.31 to 1.08)		0.51 (0.12 to 0.91)		0.49 (0.10 to 0.88)	
4-5 h	0.43 (0.17 to 0.69)		0.42 (0.13 to 0.72)		0.46 (0.16 to 0.75)		0.45 (−0.21 to 1.10)		0.12 (−0.54 to 0.78)		0.05 (−0.60 to 0.71)	
6-8 h	0.53 (0.21 to 0.84)		0.51 (0.16 to 0.87)		0.52 (0.16 to 0.87)		0.93 (0.001 to 1.86)		0.75 (−0.18 to 1.68)		0.75 (−0.17 to 1.66)	
≥9 h	0.93 (0.22 to 1.63)		0.93 (0.22 to 1.65)		0.90 (0.19 to 1.61)		0.63 (−0.85 to 2.11)		0.14 (−1.34 to 1.61)		−0.02 (−1.49 to 1.44)	
Irritability (TIDES score)												
1 h	−0.27 (−1.16 to 0.61)	.03	−0.30 (−1.18 to 0.58)	.26	−0.08 (−0.89 to 0.74)	.14	0.30 (−0.38 to 0.98)	.78	0.11 (−0.59 to 0.80)	.91	0.17 (−0.49 to 0.84)	.83
2-3 h	−0.41 (−1.27 to 0.45)		−0.37 (−1.22 to 0.49)		−0.19 (−0.97 to 0.58)		−0.03 (−0.91 to 0.85)		−0.19 (−1.08 to 0.70)		−0.32 (−1.18 to 0.55)	
4-5 h	−0.80 (−1.67 to 0.07)		−0.77 (−1.68 to 0.14)		−0.70 (−1.54 to 0.14)		0.60 (−0.90 to 2.09)		0.61 (−0.91 to 2.13)		0.46 (−1.00 to 1.93)	
6-8 h	−1.09 (−2.01 to −0.17)		−0.99 (−1.98 to 0.001)		−0.87 (−1.80 to 0.05)		−0.78 (−2.77 to 1.20)		−0.60 (−2.56 to 1.37)		−0.60 (−2.52 to 1.31)	
≥9 h	−2.35 (−4.22 to −0.49)		−1.92 (−3.81 to −0.04)		−1.92 (−3.71 to −0.12)		0.16 (−3.23 to 3.56)		−0.06 (−3.45 to 3.32)		−0.38 (−3.65 to 2.89)	
Inattention/hyperactivity (SWAN total score)												
1 h	−2.17 (−2.46 to 0.21)	.16	−1.12 (−3.49 to 1.24)	.28	−1.03 (−3.37 to 1.30)	.27	−2.66 (−4.53 to −0.80)	.05	−0.79 (−2.65 to 1.07)	.85	−0.71 (−2.54 to 1.12)	.91
2-3 h	−1.94 (−4.21 to 0.32)		−1.60 (−3.86 to 0.66)		−1.39 (−3.62 to 0.84)		−2.45 (−4.91 to 0.01)		−0.74 (−3.16 to 1.68)		−0.94 (−3.31 to 1.44)	
4-5 h	−1.32 (−3.58 to 0.95)		−2.19 (−4.57 to 0.18)		−2.25 (−4.58 to 0.09)		−0.54 (−4.72 to 3.63)		0.29 (−3.80 to 4.37)		0.14 (−3.85 to 4.14)	
6-8 h	−0.42 (−2.81 to 1.96)		−1.26 (−3.81 to 1.28)		−1.39 (−3.90 to 1.11)		−2.77 (−8.06 to 2.52)		−1.10 (−6.23 to 4.02)		−0.89 (−5.93 to 4.15)	
≥9 h	−4.65 (−9.60 to 0.30)		−4.97 (−9.90 to −0.04)		−5.16 (−10.04 to −0.29)		4.15 (−5.29 to 13.59)		3.96 (−5.13 to 13.06)		3.02 (−5.90 to 11.94)	
Inattention (SWAN inattentive subscale)												
1 h	−1.13 (−0.26 to 0.21)	.25	−0.63 (−1.97 to 0.70)	.35	−0.57 (−1.89 to 0.75)	.37	−1.42 (−2.46 to −0.37)	.08	−0.48 (−1.53 to 0.57)	.90	−0.44 (−1.47 to 0.59)	.94
2-3 h	−1.13 (−2.41 to 0.14)		−1.11 (−2.39 to 0.17)		−0.97 (−2.23 to 0.29)		−1.18 (−2.56 to 0.20)		−0.39 (−1.75 to 0.97)		−0.49 (−1.82 to 0.85)	
4-5 h	−0.72 (−1.99 to 0.56)		−1.27 (−2.61 to 0.07)		−1.27 (−2.59 to 0.05)		−0.27 (−2.61 to 2.07)		−0.07 (−2.37 to 2.23)		−0.16 (−2.41 to 2.09)	
6-8 h	−0.18 (−1.53 to 1.16)		−0.67 (−2.11 to 0.77)		−0.72 (−2.13 to 0.70)		−1.52 (−4.48 to 1.44)		−0.69 (−3.58 to 2.20)		−0.57 (−3.41 to 2.27)	
≥9 h	−1.83 (−4.63 to 0.96)		−2.10 (−4.90 to 0.70)		−2.22 (−4.99 to 0.54)		2.44 (−2.83 to 7.71)		1.67 (−3.46 to 6.80)		1.05 (−3.97 to 6.07)	
Hyperactivity/impulsivity (SWAN hyperactive/impulsive subscale)												
1 h	−1.22 (−2.51 to 0.07)	.17	−0.56 (−1.84 to 0.73)	.27	−0.51 (−1.79 to 0.76)	.27	−1.27 (−2.29 to −0.26)	.10	−0.26 (−1.27 to 0.75)	.83	−0.22 (−1.22 to 0.78)	.89
2-3 h	−1.01 (−2.25 to 0.22)		−0.60 (−1.83 to 0.63)		−0.50 (−1.71 to 0.72)		−1.25 (−2.59 to 0.08)		−0.30 (−1.61 to 1.01)		−0.41 (−1.70 to 0.88)	
4-5 h	−0.90 (−2.14 to 0.33)		−1.10 (−2.38 to 0.19)		−1.11 (−2.38 to 0.16)		−0.32 (−2.59 to 1.94)		0.36 (−1.85 to 2.57)		0.30 (−1.87 to 2.48)	
6-8 h	−0.53 (−1.83 to 0.77)		−0.72 (−2.10 to 0.66)		−0.78 (−2.14 to 0.58)		−1.44 (−4.32 to 1.44)		−0.48 (−3.26 to 2.31)		−0.36 (−3.11 to 2.39)	
≥9 h	−3.15 (−5.86 to −0.45)		−2.98 (−5.67 to −0.30)		−3.05 (−5.71 to −0.39)		2.39 (−2.71 to 7.49)		2.92 (−1.97 to 7.82)		2.41 (−2.42 to 7.24)	

Abbreviations: ASD, autism spectrum disorder; GAD-7, Generalized Anxiety Disorder Scale; PHQ-8, Patient Health Questionnaire; RCADS-P, Revised Children’s Anxiety and Depression Scale–Parent Version; SCARED, Screen for Child Anxiety Related Disorders; SWAN: Strengths and Weaknesses of Attention-Deficit/Hyperactivity Disorder Symptoms and Normal Behavior Scale; TIDES, The Irritability and Dysregulation of Emotions Questionnaire.

^a^ The reference group was 0 to 30 minutes per day.

^b^ Adjusted for child age (measured at mental health outcomes), child sex, child ancestry, family income, previous ASD diagnosis, calendar date, GAD-7, and PHQ-8.

^c^ Adjusted for child age (measured at mental health outcomes), child sex, child ancestry, family income, previous ASD diagnosis, calendar date, GAD-7, PHQ-8, and previous mental health (depression, anxiety, obsessive compulsive disorder, and attention-deficit/hyperactivity disorder) diagnosis.

^d^ The Bonferroni-corrected α is .0125 after adjusting for multiple (4) comparisons.

Higher levels of daily video game time (Table 4) were associated with higher levels of depression symptoms. There was sufficient evidence to conclude the associations with symptoms of irritability, inattention, hyperactivity, and hyperactivity/impulsivity, with the effect sizes of these associations increasing with higher video game time.

The results for electronic learning and mental health domains are shown in Table 5. High electronic-learning time was associated with higher levels of symptoms of depression and anxiety in children and youth. There was insufficient evidence to conclude the associations with symptoms of irritability, inattention and hyperactivity, inattention, and hyperactivity/impulsivity in the adjusted models.

No protective associations between video chatting and mental health symptoms across domains were identified during the COVID-19 pandemic (Table 5). Higher levels of video-chatting time per day were associated with higher levels of depression symptoms. There was insufficient evidence to conclude the associations with the rest of the mental health domains in the adjusted models.

Most of the associations persisted in the post hoc model, which was also adjusted for previous mental health diagnoses, although the associations of video game time with irritability, hyperactivity, and inattention were attenuated (Table 4 and Table 5). There was insufficient evidence that child age and sex modified the associations between screen use and mental health domains.

## Discussion

This cohort study confirms high levels of screen use among Canadian children and youth during the COVID-19 pandemic, with levels above the recommendations of no more than 1 to 2 hours of screen use per day by American Academy of Pediatrics and Canadian Paediatric Society.^40,41^ Furthermore, our study demonstrated that greater screen use was associated with higher levels of mental health symptoms in children and youth during the pandemic, consistent with evidence from pre-COVID literature.^10,11,12^ In most cases, the associations persisted even after accounting for previous mental health diagnosis.

In young children, there was evidence that higher TV or digital media time was associated with conduct problems and hyperactivity/inattention.^13,42^ The association with conduct problems is in line with previous longitudinal work also using SDQ as a tool in young children.^43^ Our finding on the association of TV or digital time with attention problems is in line with several pre-COVID longitudinal studies^44,45,46,47,48^ conducted in similar age groups, although those studies used different scales to measure inattention. Similar to previous findings,^43,46^ we did not find that TV or digital media time was associated with depression or anxiety in this age group. In this study, there was insufficient evidence of an association between video game time and any mental health symptoms in young children, consistent with previous findings.^43^ Our results could be attributed to the low exposure of video games in this age group.

In children and youth aged 6 to 18 years, higher levels of TV viewing or digital media time were associated with symptoms of depression, anxiety, irritability, and inattention. Our findings align with several pre-COVID longitudinal^49,50,51,52,53^ and cross-sectional studies^54,55,56,57^ but not with other cross-sectional studies.^58,59,60,61,62^ The discrepancy in findings may be due to differences in study design, different tools used to assess mental health, and different study population; more than one-half of this cohort had prior mental health diagnoses and could be more vulnerable to the negative effects of screen use. The positive association with depression was not observed in children with ASD. Many theories for this phenomenon have been proposed, including reduced social, cognitive, and physical demands during the pandemic; reprieve from effortful social interactions; and reduction of caregiver burden when maladaptive behaviors are present.^18^ In addition, factors such as school attendance and receiving therapies might play an important role to their functioning and emotional well-being.

There have been mixed findings examining video game time and poor mental health.^63^ Our results align with those of several prepandemic studies demonstrating that high levels of video game time were associated with depression,^52,55,59,62,64,65^ anxiety,^52,55,59,64,65^ conduct problems,^65,66^ and inattention and hyperactivity.^52,60,65,66^ Some studies, however, have also found no association^49,51,61,63^ or a negative associations^58^ between video game time and mental health symptoms; problematic gaming, rather than time spent on video games, was found to be associated with negative mental health outcomes.^63,67,68^ Most studies that found no associations used a cross-sectional design.

During the data collection period, electronic learning was the primary mode of education for children and youth in Ontario.^2^ The abrupt change in learning environment and disruption in social interactions may pose a risk to child mental health.^69,70^ A pre-COVID longitudinal study^52^ showed that electronic device use for homework had no association with children’s mental health measured. However, the definition and extent of electronic learning during COVID-19 are dramatically different from electronic learning in pre-COVID times. Research on electronic learning and mental health in children and youth younger than 18 years during COVID-19 is minimal. We identified only 1 US study,^71^ and results were consistent, showing that children aged 5 to 12 years receiving online education during COVID-19 were more likely to experience worsened mental health than children receiving in-person education.

Contrary to our hypotheses, the results of this study did not provide evidence that video chatting was protective of child mental health during COVID-19. In-person social interaction are associated with lower levels of mental health symptoms,^50,72^ yet opportunities for in-person social interaction outside of household were limited during COVID-19 because of public health restrictions^72^; therefore, digital technologies are key for children to stay connected.^73^ Results from our study showed that face-to-face interaction via digital technologies in children were not beneficial. One pre-COVID cross-sectional study^55^ showed that video chatting was associated with higher levels of symptoms of depression and anxiety in children. Similarly, a Canadian study^74^ showed that video chatting with friends during COVID-19 was associated with greater depression in adolescents.

In this study, there was no evidence that child age and sex modify the associations between screen use and mental health domains, consistent with previous pre-COVID studies.^75,76,77,78,79^ A systematic review^16^ revealed that there was inconsistent evidence of modification by child sex or age between screen use, depression and anxiety.

To our knowledge, this study is one of the first to explore the association between various types of screen use and a diverse set of mental health outcomes in children and youth during COVID-19. One key strength of this study is the use of data from 4 cohorts across a wide age range, those with and without a history of mental health conditions, and including children with neurodevelopmental problems. The use of repeated measures of exposures and outcomes, which may result in increased power and improved estimates of the strength of associations, is also a strength. Importantly, this study distinguished between different types of screen use, each shown to have different association with mental health symptoms.

Interventions from policy makers are urgently needed to promote healthful screen use and mental health in children and youth during the pandemic. These may include resuming in-person learning, reducing electronic-learning time, and encouraging children to reduce screen use. It is also important to ensure that children have access to additional mental health and social support resources and to provide teachers and therapists with training on problematic media use to help children recover from the isolation and stress of the pandemic. Although individual parents and caregivers could adopt harm reduction strategies^80^ to promote healthful screen use in children, given the unique and challenging situation of the COVID-19 pandemic, we believe that interventions will rely heavily on systemic policy changes.

### Limitations

Because this study design is testing associations, causality and directionality of results cannot be concluded. Child mental health challenges could have reversely contributed to higher screen use during the pandemic. Children with mental health symptoms might tend to be socially isolated and spend more time on screen activities than their peers, and this might be further exacerbated during the pandemic, a stressful time when few other supports were available. Residual confounding may have also occurred because of the observational nature of this study (eg, unmeasured pre-COVID child screen use, time parents spent with children, school structure and attendance, content and context of screen use, and so forth). Although we have adjusted the α for analyses involving primary outcomes, type I error may have occurred in analyses of secondary outcomes and they should be considered exploratory. We also do not have measures of total screen exposure time across all the different forms of screen use within a day, which, when combined may have compounding effects on mental health outcomes. Although we aimed to align the mental health domains across all 4 cohorts, different tools were used according to child age, which made it difficult to make direct comparison across all groups. Parent-reported data may have introduced self-reporting bias. Selection bias may be introduced by incomplete exposure and outcome data, likely because of requirements for repeated completion of multiple questionnaires. Furthermore, this study might have limited generalizability since our study population was predominantly children of European ancestry in Ontario, Canada, under specific public health guidelines.

## Conclusions

In this cohort study, different types of screen use were associated with distinct mental health symptoms in children and youth during COVID-19, suggesting that not all screen use is equal.^16^ Our findings may help inform public health guidelines that consider different forms of screen use in prevention of mental health disorders in children and youth during the pandemic. With supports from policy makers, schools and teachers, families, and health care professionals, children and youth will be better positioned to reduce screen use and promote mental health during the pandemic and beyond.

## Notes

Supplement. **eAppendix 1.** COVID-19 Public Health Measures and Daily COVID-19 Cases in Ontario, Canada **eAppendix 2.** Description of SickKids Psychiatry Cohort **eAppendix 3.** Model Equation for Linear Mixed Effects Models **eTable 1.** Child Screen Time Questions From TARGet Kids! and the 3 Cohorts (Spit for Science, SickKids Psychiatry and POND) **eFigure 1.** Sample Size Flowchart **eFigure 2.** Parent-Reported Child Daily TV/Digital Media Time, Video Game Time, e-Learning Time, and Video-Chatting Time in Younger Children (TARGetKids!) From 2020-05-21 to 2021-04-09 **eFigure 3.** Parent-Reported Child SDQ Emotional Symptoms Subscale Score in Younger Children (TARGetKids!) from 2020-05-21 to 2021-04-09 **eFigure 4.** Parent-Reported Child Daily TV/Digital Media Time, Video Game Time, e-Learning Time, and Video-Chatting Time in Older Children (Spit for Science, SickKids Psychiatry and POND Cohorts) From 2020-05-21 to 2021-04-09 **eFigure 5.** Parent-Reported Child RCADS Score and SCARED Score in Older Children (Spit for Science, SickKids Psychiatry and POND Cohorts) From 2020-05-21 to 2021-04-09 **eTable 2.** The Association Between TV/Digital Media Time and Depression in the Older Children (Spit for Science, SickKids Psychiatry and POND), Stratified by Previous ASD Diagnosis
