# Supplementary material for: Screen Use and Mental Health Symptoms in Canadian Children and Youth During the COVID-19 Pandemic
Source: JAMA Netw Open. 2021 Dec 28;4(12):e2140875. doi: 10.1001/jamanetworkopen.2021.40875 (PMC8715351; doi:10.1001/jamanetworkopen.2021.40875)
Supplement: Supplement. — eAppendix 1. COVID-19 Public Health Measures and Daily COVID-19 Cases in Ontario, Canada eAppendix 2. Description of SickKids Psychiatry Cohort eAppendix 3. Model Equation for Linear Mixed Effects Models eTable 1. Child Screen Time Questions From TARGet Kids! and the 3 Cohorts (Spit for Science, SickKids Psychiatry and POND) eFigure 1. Sample Size Flowchart eFigure 2. Parent-Reported Child Daily TV/Digital Media Time, Video Game Time, e-Learning Time, and Video-Chatting Time in Younger Children (TARGetKids!) From 2020-05-21 to 2021-04-09 eFigure 3. Parent-Reported Child SDQ Emotional Symptoms Subscale Score in Younger Children (TARGetKids!) from 2020-05-21 to 2021-04-09 eFigure 4. Parent-Reported Child Daily TV/Digital Media Time, Video Game Time, e-Learning Time, and Video-Chatting Time in Older Children (Spit for Science, SickKids Psychiatry and POND Cohorts) From 2020-05-21 to 2021-04-09 eFigure 5. Parent-Reported Child RCADS Score and SCARED Score in Older Children (Spit for Science, SickKids Psychiatry and POND Cohorts) From 2020-05-21 to 2021-04-09 eTable 2. The Association Between TV/Digital Media Time and Depression in the Older Children (Spit for Science, SickKids Psychiatry and POND), Stratified by Previous ASD Diagnosis [file jamanetwopen-e2140875-s001.pdf]

Supplemental Online Content

Li X, Vanderloo LM, Keown-Stoneman CDG, et al. Screen use and mental health symptoms in Canadian children and youth during the COVID-19 pandemic. *JAMA Netw Open*. 2021;4(12):e2140875. doi:10.1001/jamanetworkopen.2021.40875

eAppendix 1. COVID-19 Public Health Measures and Daily COVID-19 Cases in Ontario, Canada

eAppendix 2. Description of SickKids Psychiatry Cohort

eAppendix 3. Model Equation for Linear Mixed Effects Models

eTable 1. Child Screen Time Questions From TARGet Kids! and the 3 Cohorts (Spit for Science, SickKids Psychiatry and POND)

eFigure 1. Sample Size Flowchart

eFigure 2. Parent-Reported Child Daily TV/Digital Media Time, Video Game Time, e-Learning Time, and Video-Chatting Time in Younger Children (TARGetKids!) From 2020-05-21 to 2021-04-09

eFigure 3. Parent-Reported Child SDQ Emotional Symptoms Subscale Score in Younger Children (TARGetKids!) from 2020-05-21 to 2021-04-09

eFigure 4. Parent-Reported Child Daily TV/Digital Media Time, Video Game Time, e-Learning Time, and Video-Chatting Time in Older Children (Spit for Science, SickKids Psychiatry and POND Cohorts) From 2020-05-21 to 2021-04-09

eFigure 5. Parent-Reported Child RCADS Score and SCARED Score in Older Children (Spit for Science, SickKids Psychiatry and POND Cohorts) From 2020-05-21 to 2021-04-09

eTable 2. The Association Between TV/Digital Media Time and Depression in the Older Children (Spit for Science, SickKids Psychiatry and POND), Stratified by Previous ASD Diagnosis

This supplemental material has been provided by the authors to give readers additional information about their work.

© 2021 Li X et al. *JAMA Network Open.*

eAppendix 1. COVID-19 Public Health Measures and Daily COVID-19 Cases in Ontario, Canada

Starting in late March 2020, Ontario mandated closing of in-person schools, non-essential businesses, and outdoor recreation amenities including playgrounds, sports field, community gardens and exercise equipment. Restrictions in Ontario started to ease in the summer of 2020 as the province entered the recovery stage, allowing for the reopening of selected businesses and outdoor recreational facilities for team sports. In-person schools were open between September and December 2020. Provincial lockdown was issued in December 2020 as the new COVID-19 cases increased (second wave) and was phased out regionally in February and March 2021. Between April and June 2021, province-wide stay-at-home order was issued again due to the third wave of the virus. Since June 2021, Ontario has gradually reopened based on vaccination rate goals. Public health preventive measures including wearing masks, washing hands, keeping 2 metres apart from people outside of household, and vaccination have continued to be widely recommended. Daily COVID-19 cases in Ontario over time can be accessed from Enhanced Epidemiological Summary: Regional Incidence and Time to Case Notification in Ontario, Public Health Ontario (https://www.publichealthontario.ca/-/media/documents/ncov/epi/covid-19-regional-epi-summary-report.pdf?sc_lang=en.).

eAppendix 2. Description of SickKids Psychiatry Cohort

The Hospital for Sick Children (SickKids) is the largest children's hospital in Canada with over 1800 new referrals to the Department of Psychiatry annually. Children and youth (ages 4 to 17 years) referred for mental health assessment are from diverse socioeconomic backgrounds from within and outside of the Greater Toronto Area. Following referral through the centralized intake process, all children and families complete standardized measures of mental health symptoms and functional impairment and are offered the opportunity to participate in the ongoing and future research activities within the Department, including participation in a clinical research registry.

eAppendix 3. Model Equation for Linear Mixed Effects Models

Using TV/digital time and SDQ score in TARGetKids! children as an example, the model equation for the unadjusted model is:

$$\text{SDQscore}_{ijk} = \beta_0 + \beta_1 \text{TVtime}_{ijk} + \gamma_j + \alpha_i + \epsilon_{ijk}$$

Where, *j* indexes families; *i* indexes subjects within families; *k* indexes visits count within the COVID-19 study; TVtime_{ijk} is the amount of TV time in hours at visit *k* from subject *i* in family *j*; $\gamma_j \sim N(0, \sigma^2{}_j)$ represents the random effect for family *j*; $\alpha_i \sim N(0, \sigma^2{}_i)$ the random effect for subject *i*, and $\epsilon_{ijk} \sim N(0, \sigma^2)$ the residual error for visit *k* from subject *i* in family *j*.

© 2021 Li X et al. *JAMA Network Open.*

eTable 1. Child Screen Time Questions From TARGet Kids! and the 3 Cohorts (Spit for Science, SickKids Psychiatry and POND)

Questions	Response options
**TARGet Kids!:** During the past two weeks, on a typical day, how much time did your child spend:	
Watching TV or digital media (e.g., Netflix, YouTube, web surfing)?	Hours: ____ and Minutes: _____
Playing video games?	
E-learning or online schoolwork (e.g., direct instruction via Zoom or Google Meet)?	
Video-chatting/face-to-face communication (e.g., Skype, Facetime, Zoom)?	
**3 cohorts (Spit for Science, SickKids Psychiatry and POND):** Over the past two weeks, how many hours per day did your child spend:	
Watching TV or digital media (e.g., Netflix, YouTube, web surfing)?	0 -30 minutes<br>1 hour<br>2-3 hours<br>4-5 hours<br>6-8 hours<br>9 hours or more
Playing video games?	
E-learning or doing schoolwork online?	
Video chatting (e.g., Skype, FaceTime, Zoom etc.)?	

eFigure 1. Sample Size Flowchart

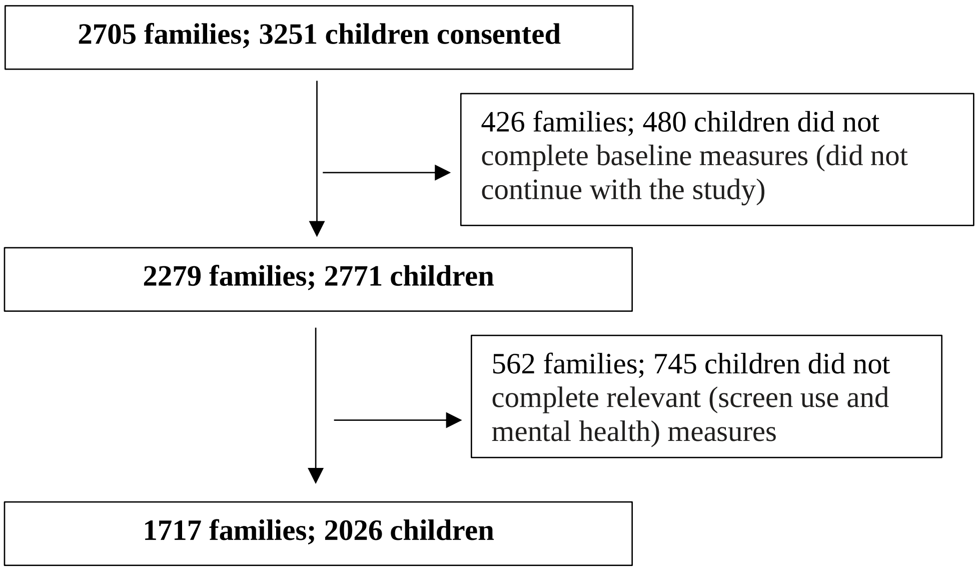

© 2021 Li X et al. *JAMA Network Open.*

eFigure 2. Parent-Reported Child Daily TV/Digital Media Time, Video Game Time, e-Learning Time, and Video-Chatting Time in Younger Children (TARGetKids!) From 2020-05-21 to 2021-04-09. The solid black line represents a 5-knot restricted cubic spline with corresponding 95% confidence bands in grey.

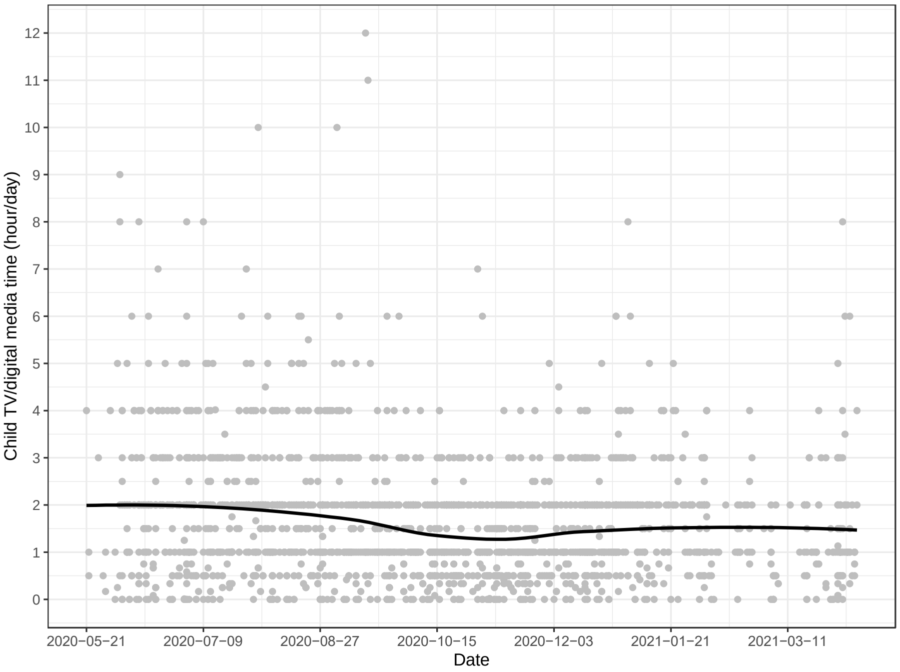

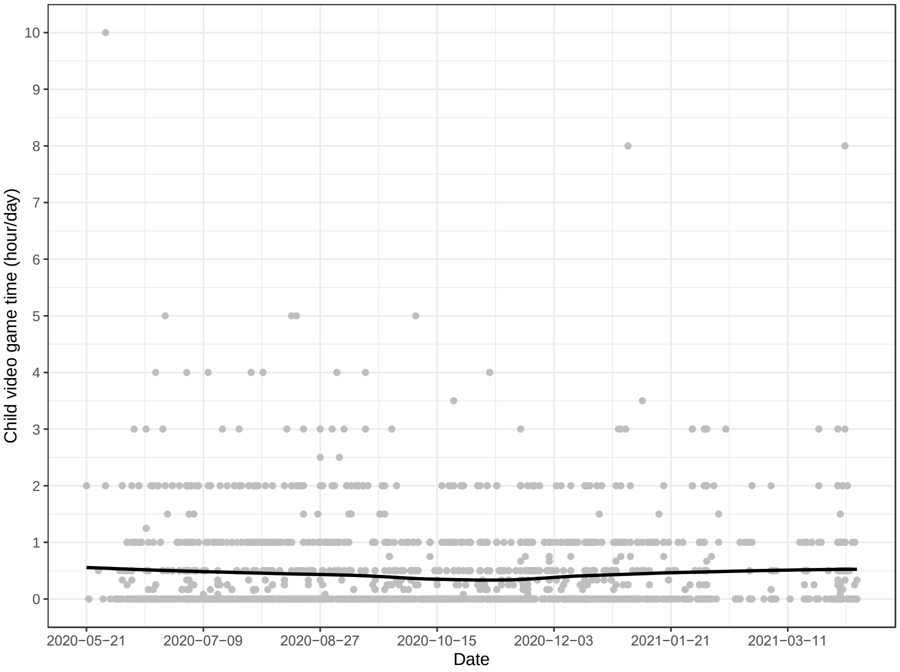

© 2021 Li X et al. *JAMA Network Open.*

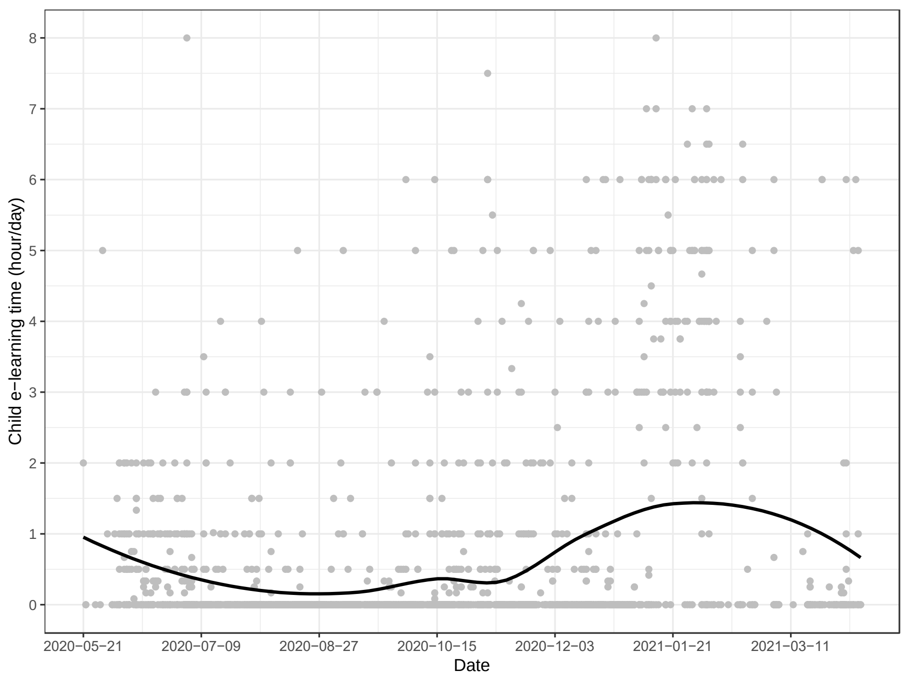

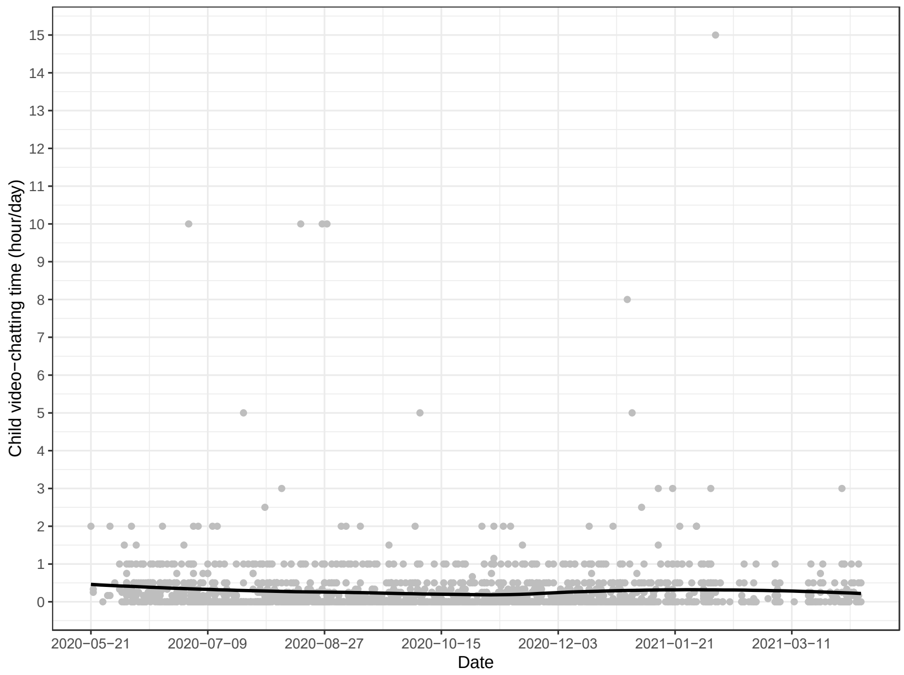

© 2021 Li X et al. *JAMA Network Open.*

eFigure 3. Parent-Reported Child SDQ Emotional Symptoms Subscale Score in Younger Children (TARGetKids!) from 2020-05-21 to 2021-04-09. The solid black line represents a 5-knot restricted cubic spline with corresponding 95% confidence bands in grey.

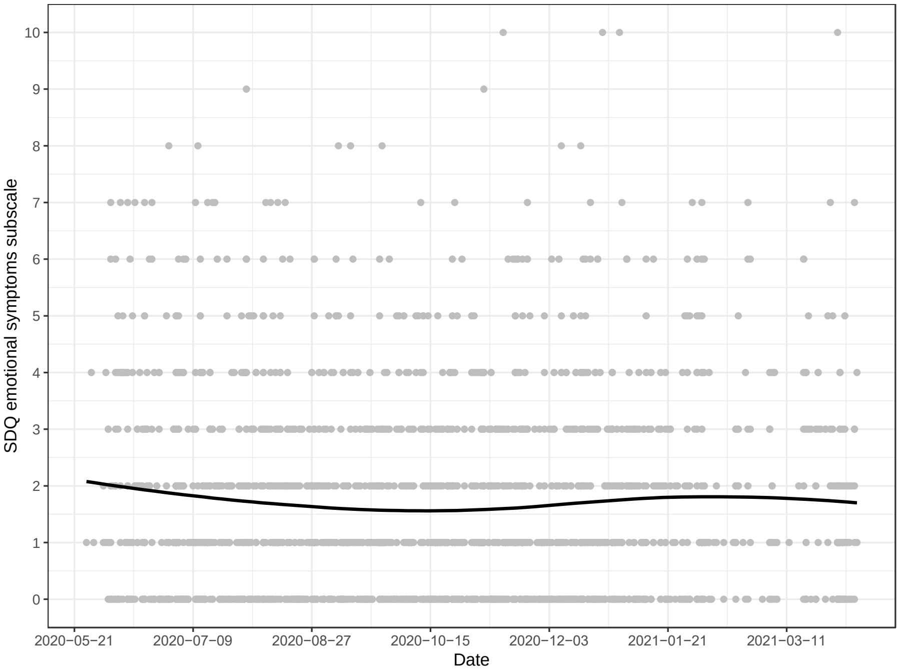

© 2021 Li X et al. *JAMA Network Open.*

eFigure 4. Parent-Reported Child Daily TV/Digital Media Time, Video Game Time, e-Learning Time, and Video-Chatting Time in Older Children (Spit for Science, SickKids Psychiatry and POND Cohorts) From 2020-05-21 to 2021-04-09. The solid black line represents a 5-knot restricted cubic spline with corresponding 95% confidence bands in grey.

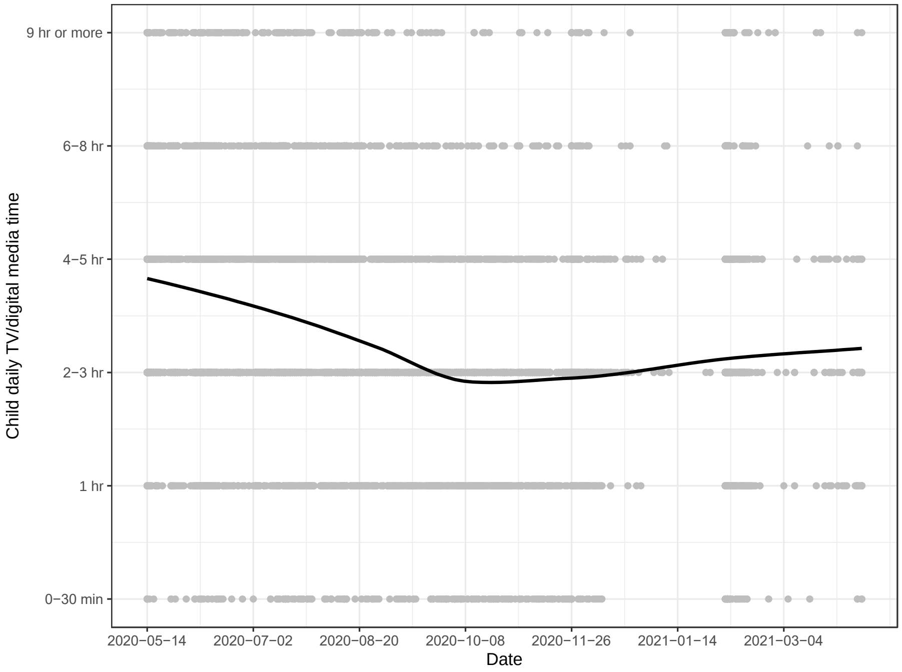

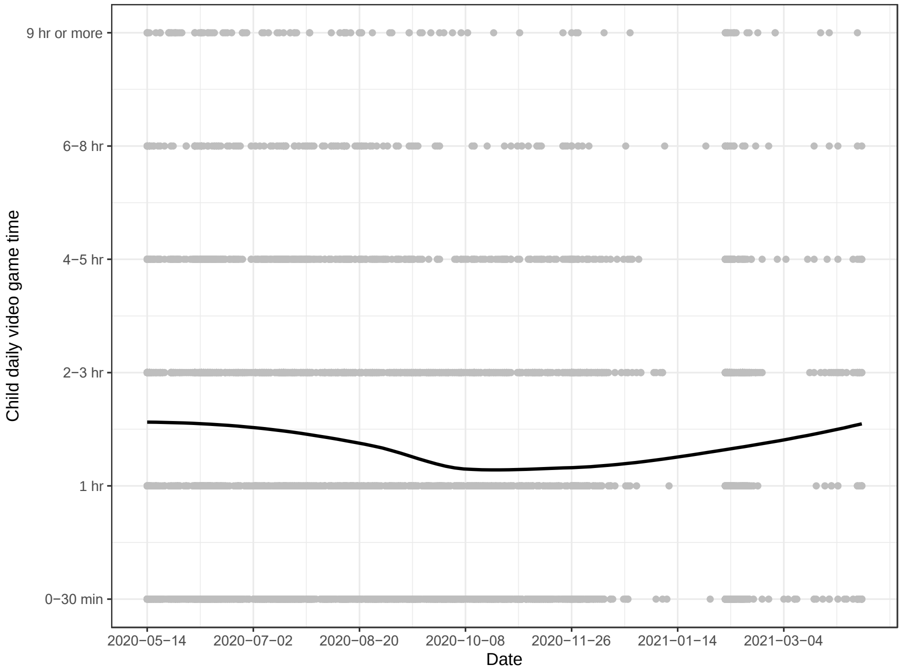

© 2021 Li X et al. *JAMA Network Open.*

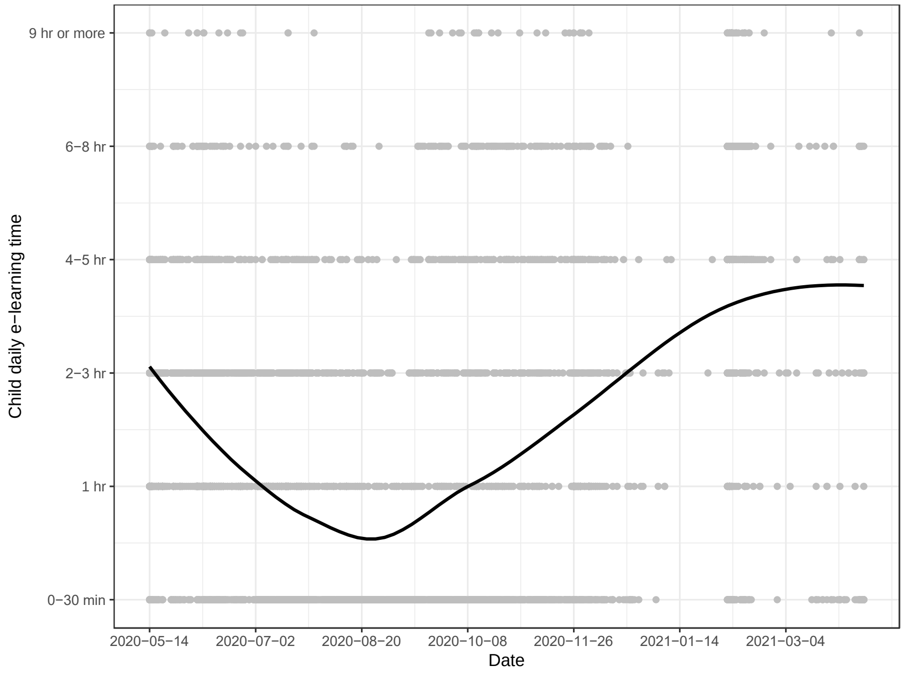

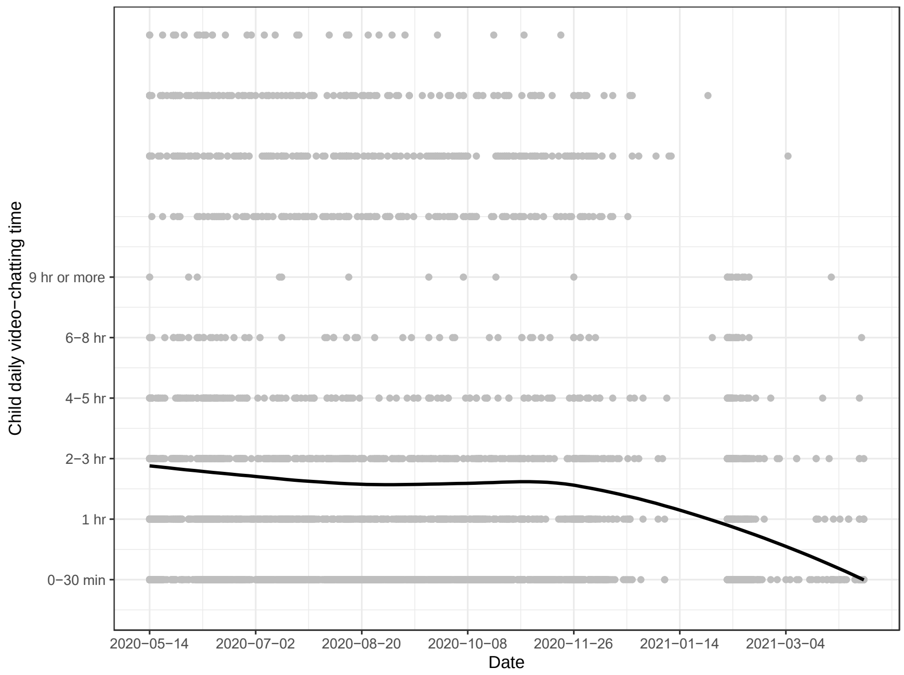

© 2021 Li X et al. *JAMA Network Open.*

eFigure 5. Parent-Reported Child RCADS Score and SCARED Score in Older Children (Spit for Science, SickKids Psychiatry and POND Cohorts) From 2020-05-21 to 2021-04-09. The solid black line represents a 5-knot restricted cubic spline with corresponding 95% confidence bands in grey.

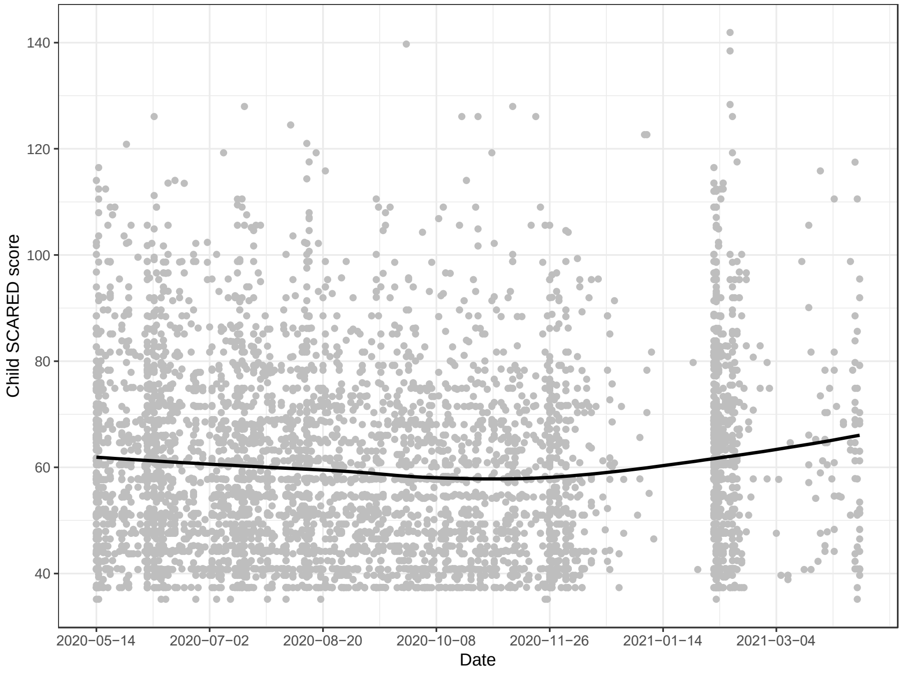

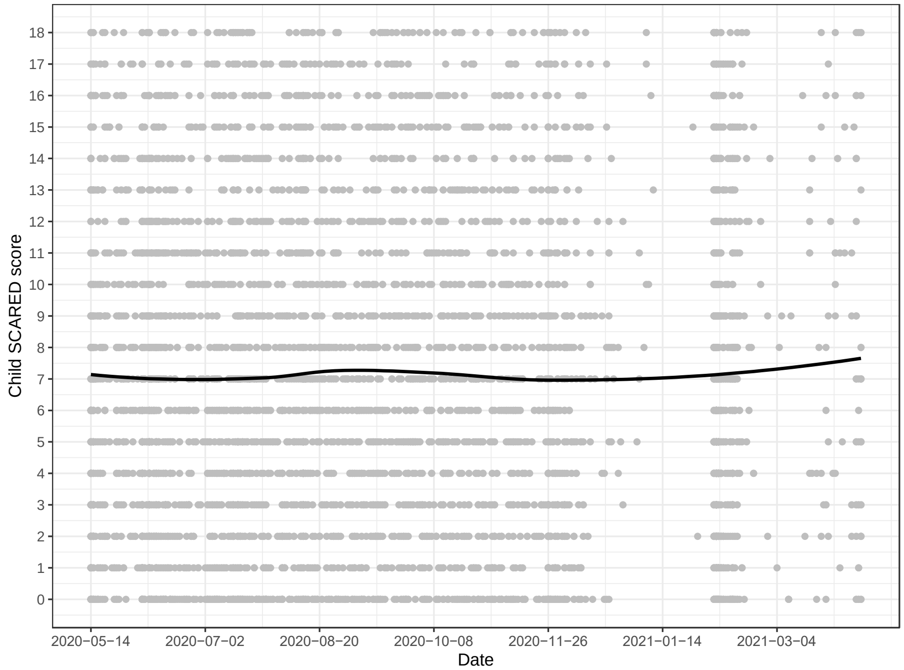

© 2021 Li X et al. *JAMA Network Open.*

eTable 2. The Association Between TV/Digital Media Time and Depression in the Older Children (Spit for Science, SickKids Psychiatry and POND), Stratified by Previous ASD Diagnosis [a]

Depression (RCADS-P T-score)				
TV/digital media time/day	Unadjusted		Adjusted [a]	
	β (95% CI)	*p*	β (95% CI)	*p*
**Diagnosed with ASD (N=237, 801 observations)**				
**1 hour**	-4.92 (-9.79 to -0.04)	.008	-4.95 (-9.81 to -0.08)	.005
**2-3 hours**	-1.31 (-5.99 to 3.38)		-1.19 (-5.86 to 3.48)	
**4-5 hours**	-0.99 (-5.82 to 3.84)		-0.98 (-5.83 to 3.86)	
**6-8 hours**	1.76 (-3.26 to 6.79)		1.63 (-3.47 to 6.73)	
**9 hours or more**	-2.20 (-7.54 to 3.13)		-2.30 (-7.84 to 3.23)	
**Not diagnosed with ASD (N=1257, 3885 observations)**				
**1 hour**	1.23 (-0.36 to 2.83)	<.001	0.87 (-0.68 to 2.43)	<.001
**2-3 hours**	3.07(1.46 to 4.70)		2.10 (0.51 to 3.69)	
**4-5 hours**	4.91 (3.17 to 6.65)		3.15 (1.41 to 4.89)	
**6-8 hours**	7.49 (5.53 to 9.45)		5.35 (3.37 to 7.34)	
**9 hours or more**	10.03 (7.68 to 12.38)		7.65 (5.32 to 9.99)	

[a] 0-30 minutes was the reference group.
[b] Adjusted for child age (measured at mental health outcomes), child sex, child ethnicity, family income, calendar date, GAD-7, PHQ-8

Abbreviations: ASD, Autism Spectrum Disorder; CI, confidence interval; RCADS-P, Revised Children's Anxiety and Depression Scale – Parent Version; GAD-7, Generalized Anxiety Disorder Scale; PHQ-8, Patient Health Questionnaire.

© 2021 Li X et al. *JAMA Network Open.*
